# Thickness of Adsorbed Polystyrene Layers by Ellipsometry^1^, ^2^

**DOI:** 10.6028/jres.067A.045

**Published:** 1963-10-01

**Authors:** Robert R. Stromberg, Elio Passaglia, Daniel J. Tutas

## Abstract

The adsorption of polystyrene from cyclohexane below the theta temperature onto chrome ferrotype plate was studied by means of ellipsometry (polarization spectrometry). In this technique changes in the state of polarization of polarized light are measured upon reflection from a film-covered surface. The measurements were carried out in situ and permitted determination of the thickness and refractive index of the swollen polymer film at the solidsolution interface. A concentration range of 0.18 to 9.7 mg/ml was studied for polymer with a molecular weight of 76,000. The thickness of the adsorbed film increased with increasing solution concentration, reaching a plateau for most of the concentration range studied. The average thickness at this plateau was approximately 210 Å. The adsorbed film was highly swollen, consisting of about 12 g/100 ml of polymer for most of the concentration range. The amount adsorbed was determined to be approximately 2.25×10^−4^ mg/cm^2^ at the plateau. Comparison of the radius of gyration of polystyrene in solvent is made to the results obtained.

## 1. Introduction

One important aspect concerning the adsorption of polymers from dilute solution onto solid surfaces that has not yet been resolved is the conformation of the polymer molecule at the interface. Early experiments on the adsorption of polymers on solid surfaces indicated that the entire polymer molecule did not contact the surface. It was proposed that the polymer is attached at a number of locations along the chain, joined by loops extending into the solution [1].^3^ This model has been widely accepted, but the number and sizes of the attached portions of the polymer chain and the sizes of the loops have not been determined.

The theoretical treatment developed by Simha, Frisch, and Eirich [2] for the adsorption of flexible macromolecules predicts a molecular conformation characterized by attachment of the molecule at relatively few locations and long chain loops extending into the solution. The sizes of these loops increase with the square root of the molecular weight. A different theoretical treatment has recently been published by Silberherg [3]. A conformation is predicted in which short stretches of segments are attached to the adsorbent surface, connected by short loops extending into the solution. The lengths of these loops are independent of the molecular weight. The shape of the molecule at the interface according to this latter treatment is dependent only on the adsorption energy and certain steric factors.

Two rather widely divergent theories, therefore, have been advanced. One results in a description of polymer adsorption characterized by relatively few attachments per polymer molecule and a rather thick adsorbed layer of what is probably a very highly solvated polymer, attached to the surface. The other leads to a film that would be of much higher density, relatively close to the surface, with many attachments per polymer molecule, thus allowing the molecule to uncoil on the surface from its conformation in the solution.

The experimental evidence concerning the thickness and conformation of the attached polymer layer is also conflicting. The apparent reduction in the diameters of fine capillary viscometer tubes has been attributed to adsorption of polymers on the walls and the thickness of the adsorbed polymer film has been calculated from such measurements [4, 5, 6, 7, 8]. These studies all indicate a thick polymer film. Adsorption studies of polymers such as poly-(vinyl acetate) on metal oxide surfaces have shown that sufficient polymer is adsorbed to indicate a thick film [9]. It was estimated that enough polymer was adsorbed to correspond to a film 10 to 40 molecules thick if the molecules were to lie flat. Application of the Simha, Frisch, and Eirich theory to the adsorption of rubber onto carbon black indicated only a few attachments per molecule [10].

Other measurements, however, have indicated that the polymer molecule may be much more closely associated with the adsorbent surface, resulting in much thinner films. Surface potential measurements on the adsorption of poly (vinyl acetate) on chrome ferrotype surfaces have indicated that the polymer uncoils almost completely until a monolayer is formed, resulting in a rather thin film [11]. Once this layer is formed it was postulated that additional polymer is deposited to build a thicker layer. This finding was supported by rate experiments with the same type of polymer and surface [12]. The adsorption of polyesters on polar surfaces such as glass and silica showed that relatively small amounts were adsorbed for these systems, corresponding to 2 to 5 layers on the glass, depending on the solvent used, and to one layer on the silica, if the polymer molecule were considered to lie flat [13]. A study of the adsorption of butyl rubber and polyisobutylene on carbon black led the investigators to the conclusion that both long and short polymers lie flat on the external surface of the carbon black [14]. Infrared spectrophotometry was used in a more direct approach to the measurement of the number of poly (alkyl methacrylate) units on silica [15]. It was reported that a relatively large number of groups were attached to the silica, inferring a relatively flattened molecule.

Thickness measurements carried out by the same authors by a sedimentation velocity method gave a film thickness of about 25 Å for one polymer and 210 Å for another. Experimental data on the adsorption of polystyrene on carbon [16] appeared to fit a simplified isotherm of Frisch and Simha [17] better if the number of anchor segments per polymer molecule was chosen to be 50 rather than 1, again inferring a flattened molecule.

The present paper reports the results of a study of the thickness and refractive index in situ of the layer of polystyrene adsorbed on chrome surfaces from cyclohexane. The measurement of the thickness and refractive index was carried out by the technique of ellipsometry (polarization spectrometry). From the refractive index the concentration of polymer in the swollen film can be calculated, and from this and the thickness, the amount of polymer adsorbed per unit area is obtained.

## 2. Measurement

### 2.1. Theory

In many ways ellipsometry is a very suitable technique for the measurement of the thickness and refractive index of an adsorbed film. Under the correct experimental conditions it is possible to measure the thickness of a thin film to within a few Angstrom units and at the same time determine the refractive index of that film to the third decimal place. It is also possible to carry out these measurements on the adsorbed film over a period of time while the film is in its swollen state in contact with the solution. Unfortunately, as will be seen below, when the refractive index of the film is close to that of the solution, the experimental precision is lowered.

The basic principles of ellipsometry are based on the original equations of Drude [18] and have been reviewed by Winterbottom [19]. Although the actual measurements with the ellipsometer are not particularly difficult, the calculations required for an exact solution of the equations are complex and very lengthy. Most of the work reported to date on the ellipsometer has been carried out using either some approximation to the solution of the equations or, more frequently, some empirical calibrations such as step wedges of barium stearate—stearic acid. Neither of these approaches was suitable to the problem of polymer adsorption. Therefore, a computational method that permitted the use of the exact equations was developed and programmed for an electronic computer [20]. The ease of the computations permitted determination of the optical constants of the specific adsorption surface immediately prior to adsorption of polymer, thus permitting increased accuracy in the determination of the properties of the films.

The method of ellipsometry is based on the measurement of changes in the state of polarization of light upon reflection from a surface. The pertinent equations are well known [18, 19] and the method has recently been reviewed [20], so that only enough detail will be given here to make the method clear.

For the purpose of analysis of reflection, the polarization vector of the light is resolved into components in the plane of incidence and in the normal to the plane of incidence (the plane of the surface). Upon reflection from the surface, the relative amplitudes and phases of the two components will be changed, so that incident linearly polarized light will be reflected in general as elliptically polarized light. For a bare surface, (i.e., one with no film) the reflection coefficient for the component in the plane of incidence, *r^p^*, and for the component in the plane of the surface, *r^s^*, are given by,

$$\begin{array}{l} r^{p}=\frac{n_{2}\cos\varphi_{1}-n_{1}\cos\varphi_{2}}{n_{2}\cos\varphi_{1}+n_{1}\cos\varphi_{2}}\\ r^{s}=\frac{n_{1}\cos\varphi_{1}-n_{2}\cos\varphi_{2}}{n_{1}\cos\varphi_{1}+n_{2}\cos\varphi_{2}} \end{array}\}$$ (1)

where *n*_2_ is the refractive index of the surface, *n*_1_ is the refractive index of the surrounding medium, and *φ* is the angle of incidence. For a metal, *n*_2_ is complex. The ratio of the reflection coefficients, *ρ*, is the basic equation of ellipsometry,

$$\rho =\frac{r^{p}}{r^{s}}=\tan\psi e^{i\Delta }$$ (2)

in which tan *ψ* is the relative amplitude reduction and Δ is the relative phase difference of the two components.

The ellipsometer measures Δ and *ψ.* From these measurements, the real and imaginary parts of the complex refractive index of the surface medium may be calculated.

For a film-covered surface, the total reflection coefficients, *R^p^* and *R*^s^, are given by

$$\begin{array}{l} R^{p}=\frac{r_{12}^{p}+r_{23}^{p}\exp D}{1+r_{12}^{p}r_{23}^{p}\exp D}\\ R^{s}=\frac{r_{12}^{s}+r_{23}^{s}\exp D}{1+r_{12}^{s}r_{23}^{s}\exp D} \end{array}\}$$ (3)

where *r*_12_ and *r*_23_ are the reflection coefficients at the film-medium and film-substrate interfaces respectively, and *D* is given by

$$D=-4\pi in_{2}\cos\;\phi_{2}\;d_{2}/\lambda$$ (4)

where *n*_2_ is the refractive index of the film, *d*_2_ its thickness, *ϕ*_2_ the angle of incidence on the substrate surface, and *λ* is the wavelength of light. The ratio of reflection coefficients is again represented by

$$\rho =\frac{R^{p}}{R^{s}}=\tan\psi e^{i\Delta }.$$ (5)

It is clear that *ψ* and Δ will be complex functions of *n*_1_, *n*_2_, *n*_3_, *d*_2_, *λ*_1_ *ϕ*_1_, and *ϕ*_2_. In principle, if all these parameters are known except *n*_2_ and *d*_2_,these may be calculated from the observed values of *ψ* and Δ, when *n*_2_ is real.

The method in principle is as follows. By measurement of *ψ* and Δ on a bare surface under a liquid, *n*_3_ (complex for a metal) is determined. Adsorption is carried out on the surface, and Δ and *ψ* measured again. By the use of eq (5)*n*_2_ and *d*_2_ can be obtained as described in the section, ‘‘Computational Method.” The ellipsometer does not measure *ψ* and Δ directly; they are, however, easily obtained from instrumental readings (20).

### 2.2. Multiple Reflections

The sensitivity and accuracy of ellipsometry is dependent upon the refractive index differences between the film and the surrounding medium [20]. The closer the refractive index of the film to that of the immersion medium, the larger the range of thicknesses and refractive indexes that will fit within experimental error. This is a very important consideration and limitation to the study of adsorbed polymer films in situ, for the film can be expected to be highly swollen with solvent and have a refractive index near that of the solvent. However, multiple reflections may be used in order to improve the sensitivity of the measurements. Although ellipsometry has in general been restricted to one reflection, the use of 8 reflections for the measurement of the adsorption of water vapor on metals in a gaseous environment has been reported [21].

The reflection coefficient for *n* reflections is

$$\rho_{n}=\rho^{n}$$ (6)

where *ρ* is the reflection coefficient for a single reflection. From eq (5)

$$\rho_{n}={\left(\tan\psi \right)}^{n}e^{in\Delta }$$ (7)

where *ψ* and Δ are the values used in eq (5), and hence

$$\tan\psi ={\left(\tan\psi_{n}\right)}^{1/n}$$ (8)

and

$$\Delta =\frac{\Delta_{n}}{n}.$$ (9)

The values of *ψ* and Δ are determined directly from the measured values of *ψ_n_* and Δ*_n_* by eqs (8) and (9), respectively.

It is assumed that the error in measuring Δ*_n_* and *ψ_n_* is a constant independent of the number of reflections, and experimentally this appears to be the case for not too many reflections. If the error in Δ*_n_* is *δ*Δ, it is clear from eq (9) that the error in the Δ used in the computations is $\frac{\delta \Delta }{n}$ if measurements are made with *n* reflections.

From the error in *ψ*, it may readily be shown that

$$\delta \psi =\frac{1}{n}\frac{\left[{\left(\tan\psi \right)}^{2n}+1\right]{\left(\tan\psi \right)}^{1-n}}{\tan^{2}\psi +1}\delta \psi_{n}.$$ (10)

It might be mentioned that for total internal reflection, tan *ψ* is unity and this expression reduces to

$$\delta \psi =\frac{1}{n}\delta \psi_{n}.$$ (11)

Therefore, for this special case as much improvement in precision is effected in *ψ* as in Δ by multiple reflections. For the more normal case of reflection from a metallic surface, tan *ψ* is approximately ½, and we obtain

$$\delta \psi =\frac{1}{5n}\frac{1+2^{2n}}{2^{n-1}}\delta \psi_{n}.$$ (12)

For a large number of reflections it is apparent that the error in *ψ* increases, i.e., *δψ*_1_ becomes less than *δψ_n_*, and multiple reflections become a hindrance rather than a help. However, for three reflections, *δψ*_1_≅*δψ*_3_, and nothing is lost. Moreover, the precision of Δ is improved by a factor of 3. For the type of surfaces used here, *ψ* is less sensitive to film thickness than is Δ, so that the latter is the more critical quantity. For these reasons three reflections was the optimum number for the type of measurements carried out here. All the work reported in this paper was carried out with three reflections.

### 2.3. Computational Method

Equation 5 cannot be solved in a closed form for the refractive index, *n*, and the thickness, *d.* The solution of this equation is described in detail in reference 20. Equation 5 may be recast into the form,

$$C_{1}{\left(\exp\;D\right)}^{2}+C_{2}\left(\exp\;D\right)+C_{3}=0$$ (13)

where *D* is given by eq (4), and *C*_1_, *C*_2_, and *C*_3_ are complex coefficients, containing as parameters all the experimental quantities, including Δ and *ψ.*

All these parameters are known, except for *n*_2_, the refractive index of the film. If a value for *n*_2_ is assumed, eq (13) may be solved to yield two values of exp *D.* From these two values, eq (4), and the assumed value of *n*_2_, two values of *d*, the film thickness are obtained. If the assumed value of *n*_2_ is not the “correct” value, both these calculated values of *d* will be complex. If the assumed value of *n*_2_ is the “correct” value, one of these thicknesses will have no imaginary part. This thickness and the corresponding refractive index are taken to be the “true” values of the thickness and refractive index of the film.

The procedure, then, is as follows. A value of *n*_2_ is assumed, and using it a value for *d* is calculated. If this is complex, another value of *n*_2_ is assumed and the calculation repeated. This is continued until a real value of *d* is obtained. On an electronic computer the procedure is quite simple.

In practice, errors in the measured values of Δ and *ψ* will cause uncertainty in both *n*_2_ and *d.* This is handled in the following manner. When a complex value of *d* is calculated as above from an assumed value of *n*_2_, the imaginary part is discarded and the real part used to calculate values of Δ and *ψ* (Δ_cal_ and *ψ*_cal_), from eq (5). These values of Δ_cal_ and *ψ*_cal_ are then compared to the experimentally observed values of Δ and *ψ*, (Δ_exp_ and *ψ*_exp_). In general, there will be a difference between the calculated and experimental quantities since the imaginary part of the complex thickness was discarded. This procedure is continued until the difference between the experimental and calculated values of Δ and *ψ* is within preassigned error limits. The corresponding range of values of *n*_2_ and *d* are taken to be the possible range of refractive index and thickness for the film. The values of *n*_2_ and *d* for which the difference between the calculated and experimental values of Δ and *ψ* is zero will be called here the “best-fit” values.

The error limits, as determined from numerous experiments, were found to be ±0.02° for the measurement of *ψ* and ±0.04° for Δ. The use of triple reflection lowers the error in Δ to 0.013° and does not affect the error in *ψ.* However, even with these reduced error limits, the range of uncertainty in both *n*_2_ and *d*, due to the small differences in refractive index between that of the film and that of the polymer solution, is still significant for the problem studied here.

## 3. Experimental Procedure

### 3.1. Materials

The polystyrene used was kindly supplied by Dr. H. W. McCormick of the Dow Chemical Company and had been prepared by the anionic polymerization of styrene. (Dow’s sample No. S102, $\bar{M}\text{w}/\bar{M}\text{n}=1.05$). For most of the work reported here the polymer described above was fractionated by conventional precipitation methods to remove any possible high and low molecular weight “tails.” The molecular weight of the fractionated polymer determined by intrinsic viscosity was 76,000, using the relation log [*η*] = −4.021 + 0.744 logM [22]^4^. Some of the work reported here was carried out using the unfractionated polymer as received. The results using this unfractionated polymer will be so labeled when discussed. The molecular weight of the polymer as determined by Dow was $\bar{M}\text{w}=82,500$, $\bar{M}\text{n}=78,500$ [23].

The solvent, cyclohexane, was freshly distilled prior to use. Measurements were carried out in a temperature-controlled room maintained at 24 °C, which is 11 degrees lower than the Flory theta temperature for this system. The concentrations studied ranged from 0.18 to 9.7 mg/ml.

### 3.2. Surface Preparation

The adsorption experiments were carried out on highly reflecting chrome surfaces. The samples themselves were 1×2 cm rectangles cut from commercial ferrotype plate. These were cleaned by immersion in warm sulfuric acid-chromic acid cleaning solution, followed by thorough rinsing in hot distilled water, then by drying at 100 °C. Immediately prior to use the slides were passed three times through a gas-oxygen flame, and immersed while still warm in solvent in the adsorption cell. The entire cleaning procedure was carried out usually within an hour of use. This procedure always resulted in hydrophilic surfaces; slides that remained in the laboratory air for short periods of time soon became hydrophobic.

### 3.3. Technique

**Figure 1:**
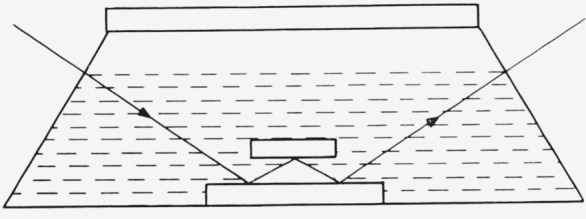
Schematic drawing of adsorption cell showing two chrome slides arranged for triple reflection of the polarized light beam.

The surfaces were prepared as described in the experimental section and placed, while still warm, into a cell containing the solvent, cyclohexane. Two slides were prepared and set in a cell as shown in figure 1. The light entered and left normal to the cell windows. The angle of incidence was 70°, the wavelength of light, 5461 Å. The upper slide was set on two gage blocks thus providing a level constant height from the lower reflecting surface. The assembly was placed on the ellipsometer stage and Δ and *ψ* determined for the triple reflection situation shown. As will be discussed later, the optical constants varied somewhat over a slide and to an even larger extent between slides. The optical constants calculated from the Δ and *ψ* determined for triple reflections were therefore an average of the optical constants for the three locations at which reflection actually took place.

After these measurements were carried out on the bare surface, the solvent was removed by hypodermic syringe and a polymer solution added. The Δ and *ψ* values for the film-covered surface were then determined as a function of time at the same locations. The determination of the properties of the adsorbed film was therefore actually a difference measurement. In the case of desorption, the solution was removed from the cell after an adsorption study means of a hypodermic syringe, and solvent added. This procedure was repeated three times and then measurements started.

## 4. Results

The thickness and refractive index of the adsorbed film are determined directly from the experimental measurements as described above. The refractive index increment, *dn/dc*, was obtained experimentally for a range of concentrations up to polymer concentrations of approximately 9 percent polystyrene in cyclohexane. The relationship between percent of polymer and refractive index was found to be linear and a value of 0.168 ml/g was obtained. Using the molar refraction relationships of Lorentz and Lorenz, and assuming additivity of specific volumes, a linear relation was also obtained for the range of swollen film concentrations studied here, resulting in a value of 0.163 ml/g. Both values were also very close to that determined experimentally [24] for a more dilute concentration range. Thus, from the refractive index of the film, the concentration of the polymer in the film can be determined. The product of this concentration and the thickness of the film gives the amount of polymer adsorbed per unit area. The experimental value 0.168 ml/g was used.

The calculations used here assume a uniform film with no refractive index gradients. This is equivalent to assuming that the polymer segment density is uniform throughout the film. This is almost certainly not the case and, in fact, Forsman and Hughes [25] have indicated that to a first approximation the segment density in the direction normal to the surface is a sum of two Gaussian curves. While it is difficult to assess the exact type of average that the assumption of a uniform film assumes, it may be seen from eq (4) that the average quantities are probably given by

$${\bar{n}}_{2}\bar{d}\cos\phi_{2}=\int_{0}^{\infty }n_{2}\left(z\right)\cos\varphi_{2}dz$$ (14)

where ${\bar{n}}_{2}$ and $\bar{d}$ are the average quantities, and *n*_2_(*z*) and cos *φ*_2_ are both functions of *z*, the distance from the surface.

**Figure 2:**
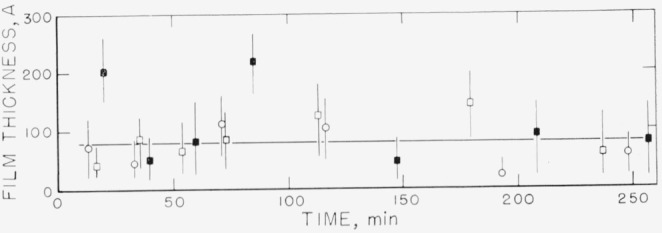
Thickness of swollen adsorbed film of polystyrene versus time of exposure to a solution for solution concentration of 0.18 mg/ml. The three different sets of points were obtained on three different sets of slides. ○, Fractionated polymer □, ■, Unfractionated polymer

**Figure 3:**
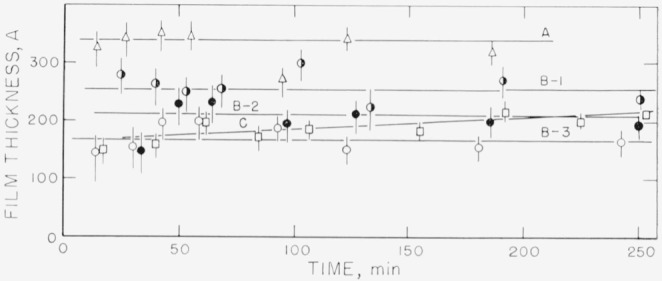
Thickness of swollen adsorbed layer of polystyrene versus time of exposure to a solution for solution concentration of approximately 8.5 mg/ml. Curves A and B obtained with fractionated polymer, curve C obtained with unfractionated polymer. Curves B–1, B–2, and B–3 obtained from measurements on different locations on the same set of slides. Curves A and C obtained from different sets of slides. Fractionated polymer; solution concentration 3.50 mg/ml. △, Curve A ◑, Curve B–1 ●, Curve B–2 ○, Curve B–3 Unfractionated polymer; solution concentration 3.32 mg/ml. □, Curve C

**Figure 4:**
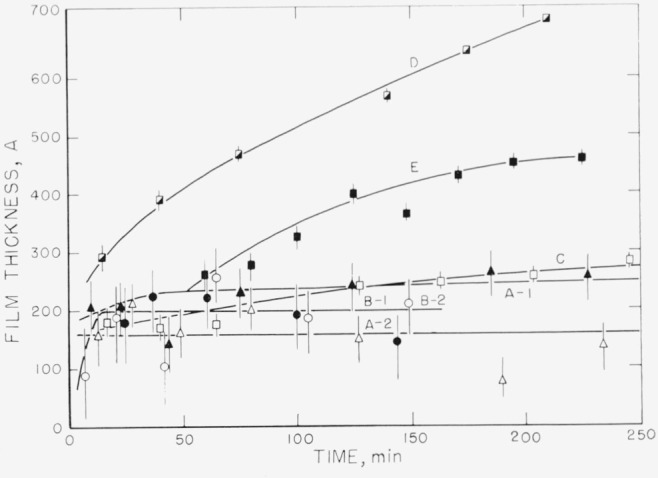
Thickness of swollen adsorbed layer of polystyrene versus time of exposure to a solution for solution concentration of 5.00 mg/ml. Curves A–1 and A–2 refer to measurements made on different portions of the same set of slides. The same notation applies to Curves B–1 and B–2. Curves A and B were obtained using fractionated samples, curves C, D, and E using unfractionated. ▲, Curve A–1 △, Curve A–2 ●, Curve B–1 ○, Curve B–2 □, Curve C ◑, Curve D ■, Curve E

**Figure 5:**
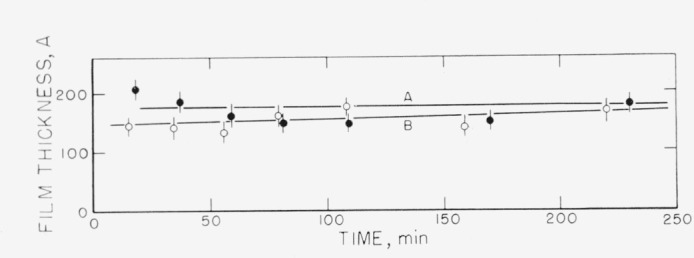
Thickness of swollen adsorbed layer of polystyrene versus time of exposure to a solution for solution concentration of 9.76 mg/ml. Both curves were obtained using fractionated polymer and each represents a different set of slides. ●, Curve A ○, Curve B

Measurements on the adsorption of polystyrene from cyclohexane solution onto the chrome surfaces were carried out for a concentration range of 0.18 to 9.7 mg/ml. There was, obviously, only negligible change in solution concentration as a result of this adsorption. Some typical individual measurements of the thickness of the swollen adsorbed layer in contact with the solution taken over a period of time are shown in figures 2 to 5. The symbols on these figures represent the “best-fit” values, while the vertical lines represent the range of thicknesses consistent with the experimental error of each individual measurement, as described in the section on Measurement.

The points shown in figure 2, obtained at a solution concentration of 0.18 mg/ml, were obtained in three runs, on three different sets of slides. Two sets were obtained using unfractionated polymer, and one using the fractionated sample. As can be observed from this figure, at this concentration there is no significant difference in the calculated thickness of the adsorbed swollen film obtained using either different sets of slides or fractionated or unfractionated polymer. The curve drawn represents an average for all the individual “best-fit” points obtained from all three runs. This average thickness is seen to be approximately 80 Å, and almost certainly less than 120 Å. The thickness did not appear to change with time over the time range studied.

The thicknesses shown in figure 3, obtained for a concentration of approximately 3.5 mg/ml (the exact concentration for each run is given in the caption for the figure) indicate a dependence of the thickness on the specific characteristics of the surface. Curves A and B were obtained using fractionated polymer, curve C using unfractionated material. Each curve for the fractionated polymers represents the average of the “best-fit” values for that particular run. Curves B–1, B–2, and B–3 are the results of measurements made at three different locations on the same set of slides. Thickness curves that differed from each other were obtained at the different locations on the same set of slides as well as on the different slides. This is taken as an indication that the individual locations studied differed from each other, perhaps in the number of adsorption sites available.

**Figure 6:**
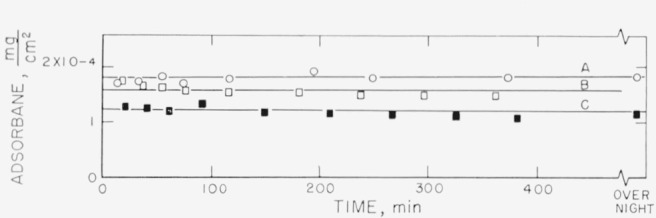
Adsorbance of polystyrene for solution concentration of 0.18 mg/ml. The symbols on this figure represent the runs using the identical symbols used in figure 2. ○, Fractionated polymer □, ■, Unfractionated polymer

**Figure 7:**
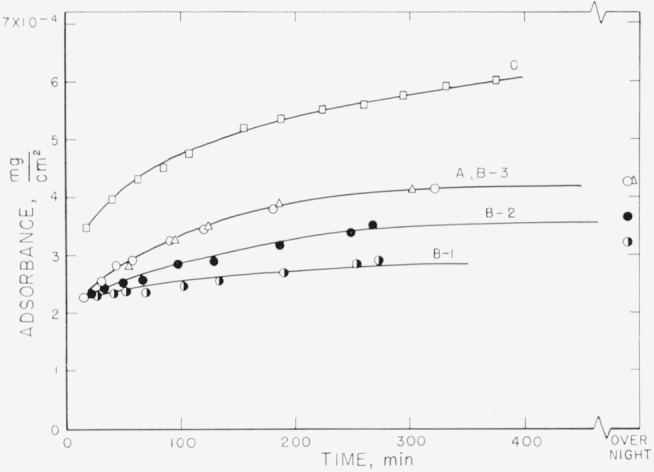
Adsorbance of polystyrene for solution concentration of approximately 3.5 mg/ml. The designation of the curves by letter and the symbols used correspond to those used in figure 3. Fractionated polymer; solution concentration, 3.50 mg/ml △, Curve A ◑, Curve B–1 ●, Curve B–2 ○, Curve B–3 Unfractionated polymer; solution concentration, 3.32 mg/ml. □, Curve C

It can be observed from figure 3 that the curve obtained with the unfractionated polymer fell within the limits of the fractionated material, although the thickness of the unfractionated material appeared to increase somewhat with time, while the other curves appear to be flat. However, a comparison with the adsorbance^5^ in figure 7 shows that more polymer was actually deposited from the unfractionated polymer, as seen by curve C. Although the swollen film thicknesses seen in figure 3 are about the same for fractionated and unfractionated polymer, the concentration of polymer in the unfractionated swollen polymer film was appreciably higher, resulting in the curve C shown in figure 7. Examination of figure 6 shows that there was no significant difference between the adsorbances for the fractionated and unfractionated samples that were measured at the lower concentration of 0.178 mg/ml.

**Figure 8:**
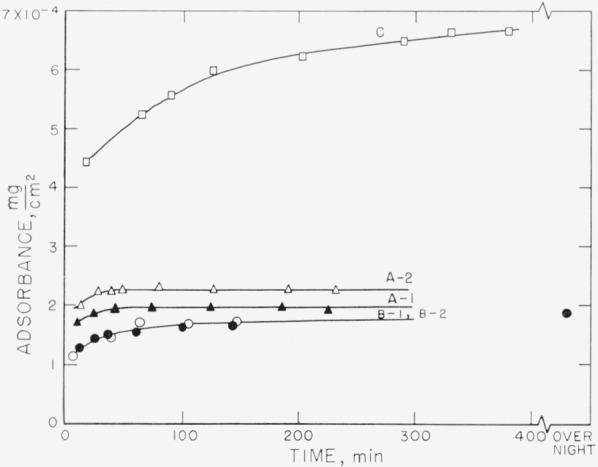
Adsorbance of polystyrene for solution concentration of 5.00 mg/ml. The designation of the curves by letter and the symbols used correspond to those used in figure 4. Curves A and B represent fractionated sample, curve C unfractionated. ▲, Curve A–1 △, Curve A–2 ●, Curve B–1 ○, Curve B–2 □, Curve C

The thickness results obtained for the concentration of 5.00 mg/ml are shown in figure 4. In this case curves A–1 and A–2 were obtained from different portions of one set of slides, and curves B–1 and B–2 from another set of slides. All four of these determinations were carried out with fractionated polymer. The averages of the “best-fit” values range from about 160 to 240 Å. In the examples shown in this figure, the differences in thicknesses measured from one location on one set of slides to another location on the same set of slides was greater than the differences from one set of slides to another. Curves C, D, and E were obtained using unfractionated polymer. Curves D and E represent thicknesses that are much greater than those obtained with the fractionated polymer and that continue to increase with time. It should be noted that the range of uncertainty is much less for the thicker films than for the thinner. Curve C is seen not to differ appreciably from those obtained with the fractionated material. The amounts adsorbed for this solution concentration (shown in fig. 8), again indicate that much more polymer is deposited from the unfractionated material, although the swollen film may have approximately the same thickness as the fractionated polymer.

Figure 5 shows the results of the thickness measurements obtained with a solution concentration of 9.7 mg/ml. In this case, both of the runs using fractionated polymer resulted in thickness measurements that were quite similar to each other. The thicknesses obtained were somewhat smaller than had been obtained with the lower concentrations. The range of uncertainty was decreased because of the increased polymer concentration in the adsorbed layer. The behavior of the unfractionated polymer at this concentration is not shown in the figure, but it was similar to that shown for the 5 mg/ml concentration in figure 4.

**Figure 9:**
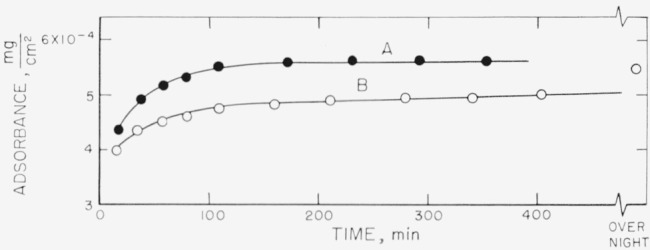
Adsorbance of polystyrene for solution concentration of 9.76 mg/ml. Both curves were obtained using fractionated polymers and the lettering of the curves and the symbols used are identical with that used in figure 5.

Figures 6 to 9 represent the individual calculated adsorbances for the same solution concentrations used for figures 2 to 5. The symbols used for the individual points and the lettering of the curves are identical on both sets of curves for the same solution concentration. The amount adsorbed is a function of the refractive index and thickness of the swollen film. As the calculated thickness of the swollen film varies with the assumed refractive indexes, the value of the adsorbance is relatively independent of the uncertainties that are inherent in the determination of the swollen film thicknesses. Differences in the amounts adsorbed for swollen films of approximately the same thickness represent, of course, different densities of the polymer films.

Figure 6 obtained for the concentration of 0.178 mg/ml, shows that for this relatively dilute concentration the adsorbance for fractionated and unfractionated polymer was virtually the same. This is probably due to the fact that there was no precipitation of unfractionated polymer. Figure 7 shows the amounts adsorbed for the solution concentration of 3.5 mg/ml. The arrangement of the curves is observed to be different from that shown in figure 3, with the quantities ranging from about 2.8×10^−4^ to 4.2×10^−4^ mg/cm^2^ for the fractionated polymer. As mentioned earlier, the quantity deposited for the unfractionated material, curve C, does not fall with the group of curves obtained for the fractionated polymer.

The adsorbances obtained for the 5 mg/ml concentration are given in figure 8, and range from about 1.8×10^−4^ to 2.3×10^−4^ mg/cm^2^. The arrangement of the curves with respect to each other is again seen to be somewhat different than that for the solvated films represented in figure 4. A more definite increase in quantity with time is observed during the early portion of the adsorption than was evident from the solvated film thicknesses. As the solution concentration increases, the difference between the fractionated and unfractionated polymer solutions becomes increasingly more apparent. Curve C is seen to be very different from the other samples, although the swollen film thicknesses were similar. The other unfractionated curves shown in figure 4 are not given but would be much greater in amounts deposited. Although the thicknesses of the swollen films for the solution concentration 9.7 mg/ml shown in figure 5 are relatively small, the quantities given in figure 9 are quite high, ranging from about 4.9 ×10^−4^ to 5.6×10^−4^ mg/cm^2^.

**Figure 10:**
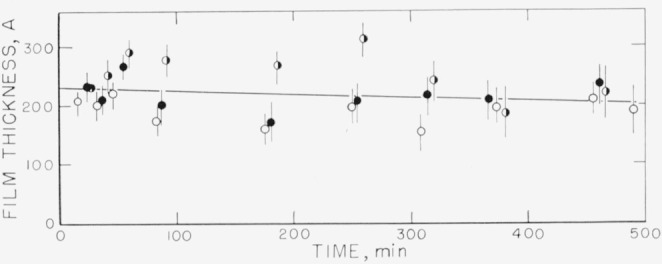
Desorption: Thickness of adsorbed polystyrene layer versus time of exposure of film to solvent. Films adsorbed from solution concentration of 3.50 mg/ml. ◑, Curve B–1 ●, Curve B–2 ○, Curve B–3

**Figure 11:**
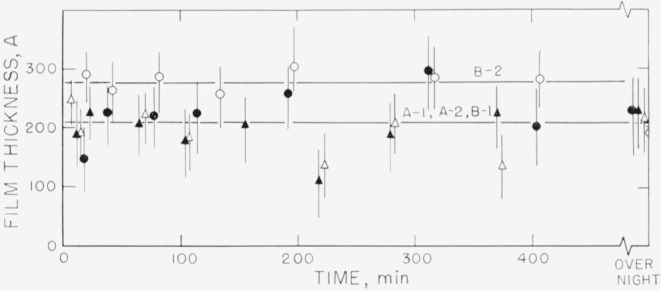
Desorption: Thickness of adsorbed polystyrene layer versus time of exposure of film to solvent. Films adsorbed from solution concentration of 5.00 mg/ml. ▲, Curve A–1 △, Curve A–2 ●, Curve B–1 ○, Curve B–2

**Figure 12:**
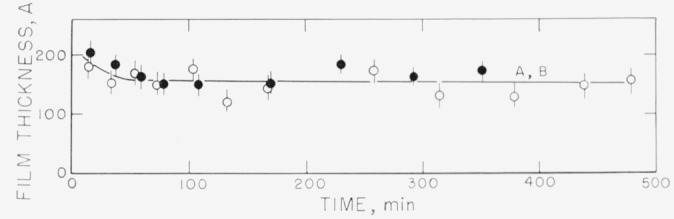
Desorption: Thickness of adsorbed polystyrene layer versus time of exposure of film to solvent. Films adsorbed from solution concentration of 9.76 mg/ml. ●, Curve A ○, Curve B

The desorption of polystyrene was studied by the same technique and resulted in swollen film thicknesses that were not appreciably different from those obtained during adsorption. Typical results are shown in figures 10, 11, and 12. Curves B–1, B–2, and B–3 in figure 10 are approximately the same thickness or only slightly less than that shown by the curves in figure 3. The desorption curves shown in figure 11 show a slightly increased thickness over those shown in the adsorption isotherms in figure 4. In the case of the most concentrated solution studied, the desorption curves shown in figure 12 are almost identical with the adsorption curves shown in figure 5.

## 5. Discussion

**Figure 13:**
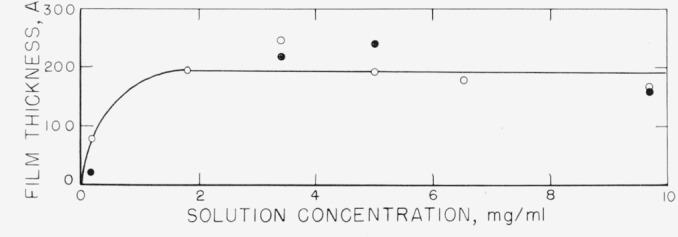
Average swollen film thickness as a function of solution concentration. The points represent the averages of several measurements. ○, Adsorption thicknesses. ●, Desorption thicknesses—film adsorbed from solution concentration shown, but measured in contact with solvent.

It was observed that there was considerable variation in the thickness of the adsorbed polymer film. It appears that for most of the range of concentrations studied more variation was caused for the fractionated polymer by the differences in the particular surface studied than by changes in solution concentration. Further, it was observed that there were no large changes in the thickness of the adsorbed film with time, several minutes being required before the initial measurements were made. If the average thicknesses for each run at a specific concentration are then averaged for that concentration, the points represented by the open circles in figure 13 are obtained. The curve drawn through these points, therefore, represents the average of the individual “best-fit” point averages for the concentration range studied. It is observed that the average thickness of the swollen polystyrene film on the chrome surface is about 200 Å for most of the concentration range given. The value of thickness obtained at the lowest concentration appears to be significantly lower than the remainder. The desorption thickness values, obtained from fewer runs, are also given, and are seen not to differ appreciably from the adsorption curve.

This work was carried out at a temperature below the theta temperature. The increased thicknesses and amounts of unfractionated polymer deposited probably are caused by precipitation of a portion of polymer with a molecular weight higher than 76,000. This is especially indicated by the increasing differences between fractionated and unfractionated polymer as the concentration increases.

**Figure 14:**
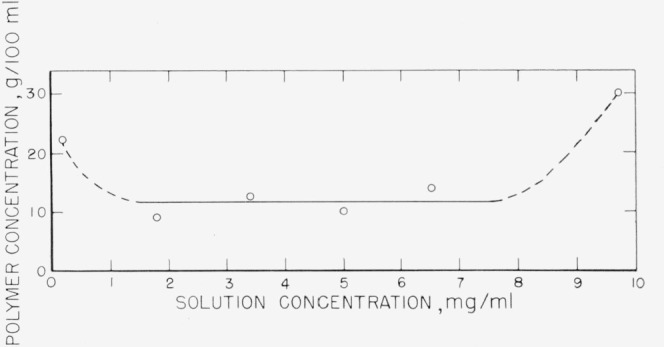
Concentration of polymer in swollen adsorbed film as a function of solution concentration. The points represent averages of several runs.

Figure 14 shows the concentration of polymer in the adsorbed film for the fractionated sample. The points are the averages of several runs, obtained in the same manner as those in figure 13. It is observed that the film contains about 12 g of polymer per 100 ml of solution for most of the concentration range. The film adsorbed from the 9.7 mg/ml concentration contained approximately 30 percent polymer. It is possible that as the solution concentration increases, a multilayer adsorption occurs. This was not evidenced by the film thickness measurements, but an intertwining of polymer chains may occur at high solution concentrations, retaining the same film thickness, but greatly increasing the amount of polymer in the layer. There is also an indication that the percentage of polymer in the film is greater at low solution concentrations. In this case, the film thickness was lower than the remainder of the concentration range and it is possible that a thinner, more tightly bound film is first formed, similar to the situation proposed by Gottlieb [11].

**Figure 15:**
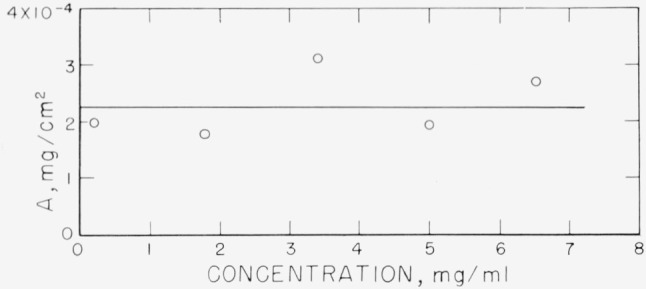
Adsorption isotherm obtained from ellipsometer data. Points represent averages of individual runs.

From the data obtained here, the amount of polymer adsorbed can be calculated and an isotherm constructed. This is shown in figure 15. Not shown on this figure is the amount of material adsorbed at the solution concentration of 9.7 mg/ml. The amount calculated for this concentration was about 5.2×10^−4^ mg/cm^2^. As discussed above, this large amount may be caused by multilayer adsorption. The remainder of the isotherm shows a plateau, extending for the entire range of concentrations studied, with the exception of the 9.7 mg/ml concentration, at about 2.25 mg/m^2^.

Although a different molecular weight polystyrene was used, the results reported here are considerably lower than the 1500 A calculated by Öhm [4] for glass, or similar large values obtained by Fendler, Rohleder, and Stuart [7]. Our results are also much lower than the effective thickness of approximately 5000 Å obtained by Tuijnman and Hermans [6] for poly (vinyl acetate) on glass, or that calculated by Takeda and Endo [8] for poly (vinyl chloride) on glass. All of these measurements were carried out by viscosity measurements and the effective thickness average obtained by such a technique is undoubtedly quite different from that obtained by the method described here, It is not expected that the differences between the chromium-chromium oxide and glass surfaces are sufficiently great to account for the large differences in the thicknesses ascribed to the film.

The possibility of a two-stage adsorption as advanced by Gottlieb [11] could not be verified or disproved. The thicknesses found here are similar to the 210 Å reported by Fontana and Thomas [15] for the adsorption of a copolymer of stearyl methacrylate and *N*-vinyl-2-pyrrolidone on silica, although they also reported a 25 Å thickness for an alkyl methacrylate on silica.

It is of interest to compare the values obtained from these measurements with the root-mean-square distance of an element from the center of gravity. The radius of gyration for polystyrene in a poor solvent is approximately 83 Å for the molecular weight used here [26]. If the amount adsorbed is taken from figure 15 to be approximately 2.25 mg/m^2^, then, assuming hexagonal packing of a “monolayer”, the centers of the molecules are calculated to be 88 Å apart. A model consistent with this dimension would be one with almost complete interpenetration of the random coils. It would be of great interest, of course, to relate the measurement of a thickness of 200 Å, as measured by the ellipsometer, to these considerations. However, until more is known about the type of average determined by the ellipsometer, and a realistic model for the adsorbed polymer molecule developed, this cannot be done.

At first glance it appears that our results are somewhat more consistent with the theoretical treatments of Simha, Frisch, and Eirich [2] than with that of Silberberg [3] who predicts a rather flat adsorbed molecule with only short loops extending into the solution for situations where there is a multitude of available sites. It seems apparent that in the system studied here, the loops are extending very far into the solution, unless there is first adsorbed a tightly bound layer and what is being observed here is some multilayer adsorption on top of this layer. However, if one considers that the adsorbent surface is a highly polar oxide surface and that polystyrene is not a very polar molecule, there would be, therefore, only relatively few sites available for adsorption, yielding another situation described by Silberberg.

## Footnotes

3 Figures in brackets indicate the literature references at the end of this paper.

4 Fractionation and molecular weight by Warren H. Grant, NBS.

5 We use the term *adsorbance* to denote the amount adsorbed per unit area, in mg/cm^2^.
